# Pediatric Clinicians’ Use of Telemedicine: Qualitative Interview Study

**DOI:** 10.2196/29941

**Published:** 2021-12-02

**Authors:** Julia B Finkelstein, Elise S Tremblay, Melissa Van Cain, Aaron Farber-Chen, Caitlin Schumann, Christina Brown, Ankoor S Shah, Erinn T Rhodes

**Affiliations:** 1 Department of Urology Boston Children's Hospital Boston, MA United States; 2 Division of Endocrinology Boston Children’s Hospital Boston, MA United States; 3 Department of Medical Informatics School of Community Medicine University of Oklahoma Tulsa, OK United States; 4 Innovation and Digital Health Accelerator Boston Children’s Hospital Boston, MA United States; 5 Department of Ophthalmology Boston Children’s Hospital Harvard Medical School Boston, MA United States

**Keywords:** pediatrics, telemedicine, video visits, communication, webside manner

## Abstract

**Background:**

Bedside manner describes how clinicians relate to patients in person. Telemedicine allows clinicians to connect virtually with patients using digital tools. Effective virtual communication or *webside manner* may require modifications to traditional bedside manner.

**Objective:**

This study aims to understand the experiences of telemedicine providers with patient-to-provider virtual visits and communication with families at a single large-volume children’s hospital to inform program development and training for future clinicians.

**Methods:**

A total of 2 focus groups of pediatric clinicians (N=11) performing virtual visits before the COVID-19 pandemic, with a range of experiences and specialties, were engaged to discuss experiential, implementation, and practice-related issues. Focus groups were facilitated using a semistructured guide covering general experience, preparedness, rapport strategies, and suggestions. Sessions were digitally recorded, and the corresponding transcripts were reviewed for data analysis. The transcripts were coded based on the identified main themes and subthemes. On the basis of a higher-level analysis of these codes, the study authors generated a final set of key themes to describe the collected data.

**Results:**

Theme consistency was identified across diverse participants, although individual clinician experiences were influenced by their specialties and practices. A total of 3 key themes emerged regarding the development of best practices, barriers to scalability, and establishing patient rapport. Issues and concerns related to privacy were salient across all themes. Clinicians felt that telemedicine required new skills for patient interaction, and not all were comfortable with their training.

**Conclusions:**

Telemedicine provides benefits as well as challenges to health care delivery. In interprofessional focus groups, pediatric clinicians emphasized the importance of considering safety and privacy to promote rapport and *webside manner* when conducting virtual visits. The inclusion of *webside manner* instructions within training curricula is crucial as telemedicine becomes an established modality for providing health care.

## Introduction

### Background

Telehealth is a broad term that describes the provision of health care remotely using technological tools with or without a video connection [1]. Telemedicine is a subset of telehealth that refers specifically to the provision of *clinical* health care services. This can involve asynchronous transmission (ie, store and forward) of information for later review by a clinician or synchronous, live conferencing [2]. In a virtual visit, the patient and clinician are connected via a live, synchronous, interactive video system.

Within pediatrics, before the onset of the COVID-19 pandemic, telemedicine had been used in a variety of specialties, including neonatology, critical care, ophthalmology, dermatology, and urology [3-6]. Although there are additional concerns and logistical issues in implementing this type of care delivery in pediatrics, several studies have also demonstrated that the use of this technology is feasible, safe, economical, and beneficial to families because of reduced absenteeism for children and their caregivers [4,7,8]. Within this context, a telemedicine program was launched at our institution, a large tertiary care pediatric center, in late 2016.

Although telemedicine was originally used to access patients in remote locations, virtual visits have increasingly been accepted as a tool to provide real-time, convenient medical care. In part, this is because of the rapid advances in technology and the widespread affordability and accessibility of basic telemedicine tools (eg, mobile devices) [9]. Since its inception in late 2016, our institutional telemedicine program rapidly expanded to include 22 departments and 2345 virtual visits (<1% of total outpatient visits) when the study was initiated in November 2018. Patients participated in the virtual visit from their home or any other convenient location. The virtual visit is the telemedicine focus in this analysis.

### Objectives

With the rapid application of this innovative technology, it is essential to preserve standards for high-quality and meaningful care. This includes effective virtual communication or good *webside manner* [10,11]. When communicating through technology, bedside manner, or the way in which clinicians relate with patients, may not be implemented in the same way as in person. Although maintaining a connection with patients and having therapeutic in-person interactions are considered *good* bedside manner, the ability to connect with patients virtually or in a webside manner is a novel concept. Modifications to the environment and clinician communication style may be necessary to build rapport and positively affect the visit experience. As virtual visits are becoming a powerful tool for clinicians to connect with children and families, it is essential that clinicians develop these skills. The aim of this study, conducted before the COVID-19 pandemic, is to understand the experiences of telemedicine providers with patient-to-provider virtual visits and communication with families at a single large-volume children’s hospital. We anticipate that these qualitative data will be useful for guiding program development and training future clinicians, information that remains salient, given the established role of virtual visits in the context of the COVID-19 pandemic.

## Methods

### Study Design

A total of 2 focus groups of clinicians performing virtual visits were conducted before the COVID-19 pandemic, with the aim of generating discussions around shared experiences in the implementation and practice of telemedicine in their individual disciplines. The institutional review board of Boston Children’s Hospital deemed this study exempt.

### Sample

A purposive sample of clinicians who performed virtual visits at a single pediatric institution was recruited to participate in the study [12]. In keeping with purposive sampling, potential participants were invited based on a desire to represent groups that were already more extensively involved in virtual visits (clinical champions), becoming more involved in virtual visits (those increasing their volume), and representing both medical and surgical specialties as well as different health care disciplines (Doctor of Medicine vs nurse practitioner, registered nurse, or physician assistant). The participants did not need a minimum number of years of experience with virtual visits, and not all clinicians were contacted. Dedicated clinical champions were working with the hospital’s virtual visit team to increase virtual visit volume and engagement in their department.

To reflect the diversity of experiences with virtual visits at the institution, groups were constructed based on specialty (both medical and surgical) and telemedicine experience (defined as the number of virtual visits conducted). At the time of the study, because of insurance restrictions, virtual visits at the institution were limited primarily to postoperative and established visits. Recruitment for the first focus group included dedicated clinical champions for the virtual visit team, and the second group included clinicians who actively increased their virtual visit volume. Clinicians were contacted via email and invited to participate in the focus groups. If willing, the clinicians completed a survey and participated in a focus group. A catered breakfast was provided to the participants.

### Interview Guide and Procedures

On the basis of a review of the literature, interprofessional collaboration, and discussion with clinicians with telemedicine experience, a clinician survey and focus group guide were developed. The survey contained a mix of 5-point Likert scales, binary (yes or no), and multiple-choice questions that covered topics including the individual clinician experience with virtual visits and their opinions on the efficacy of the visits. The semistructured focus group guide contained open-ended questions regarding clinicians’ general experience with virtual visits, impression of preparedness, strategies for establishing rapport with patients and families, and suggestions for future considerations (Multimedia Appendix 1).

The 2 focus groups were conducted to include clinicians from the following disciplines: primary care, urology, ophthalmology, gynecology, cardiac surgery, psychiatry, neurosurgery, and orthopedics. Groups were moderated by 1 team member and supported by 3 others. One of the team members was a virtual visit clinical champion and participated in the first focus group and served as an observer in the second. Clinicians participated in person or by phone, and all sessions were digitally recorded and professionally transcribed.

### Data Analysis

Field notes and focus group transcripts were reviewed to identify themes that clinicians voiced on experiences related to their virtual visit encounters. Thematic analysis is a common research strategy among qualitative researchers. It enables investigators to form generalized commentary in a subject area via the compilation of participant-level experiences and opinions [13,14]. After review and discussion, an initial set of codes was generated by the team and then applied to the transcript data. Meetings were then held to resolve any discrepancies, and a final coding framework was agreed upon. The analysis generated a clear saturation of thematic content between the 2 focus groups. After the iterative coding process was complete, the research team used NVivo 12 software (QSR International) to organize the data for further discussion. The qualitative data were then iteratively reviewed so that codes could be collated into themes and subthemes. Through this process, 3 overarching themes were identified that best described and compiled the body of data.

## Results

### Participant Characteristics and Survey Results

The focus groups were conducted in November 2018 and March 2019. A total of 11 clinicians participated in the study, who were split between the 2 focus groups of 6 (55%) and 5 (45%) participants. Approximately 73% (8/11) were physicians, and the groups were divided into medical and surgical specialties (Table 1). The focus group duration was an average of 69 (SD ± 8.5; range 63-75) minutes.

The survey results from the participants (8/11, 73%) are summarized in Table 2. All respondents answered each question.

**Table 1:** Focus group participant characteristics (N=11).

Participants		Focus group 1 (n=6)	Focus group 2 (n=5)
**Number of participants by subspecialty type, n (%)**			
	Surgical	3 (50)	4 (80)
	Medical	3 (50)	1 (20)
**Number of participants by clinician type, n (%)**			
	MD^a^	4 (67)	4 (80)
	PA^b^, RN^c^, NP^d^	2 (33)	1 (20)
**Number of virtual visits completed, range^e^**			
	MD participants	1-106	4-135

^a^MD: Doctor of Medicine.

^b^PA: physician assistant.

^c^RN: registered nurse.

^d^NP: nurse practitioner.

^e^Data for physician assistant, registered nurse, and nurse practitioner participants were not available.

**Table 2:** Clinician survey results.

Question	Answer options	Responses, median (IQR)
Approximately how many virtual visits have you completed?	Free text	50 (7.25-60.5)
How prepared did you feel to start virtual visits after virtual visit training?	Not at all Not really Neutral/I don’t know A little bit Completely	4.00 (3.75-4.25)
Generally, how satisfied have you been with the virtual visit experience?	Not satisfied Slightly satisfied Neutral/I don’t know Very satisfied Extremely satisfied	4.00 (4.00-4.25)
What measures do you take to minimize background noise or change other environmental conditions that may affect the quality of the encounter?	Wearing headphones Conducting the virtual visit in a private office or space Put a sign on my door Use partitioning wall Other, please specify_____	Conducting the virtual visit in a private office or space: 5 clinicians; Put a sign on my door: 1 clinician; Other: made sure I had a wall or normal plane behind me: 1 clinician
I am able to communicate effectively with the patient and family.	Strongly disagree Disagree Neutral/I don’t know Agree Strongly agree	4.5 (4.00-5.00)
I am able to obtain sufficient information even though the physical examination is not in-person.	Strongly disagree Disagree Neutral/I don’t know Agree Strongly agree	4.00 (3.25-4.00)
How do you see providers being educated on virtual visits in the future?	In-person training Self-paced online learning Interactive simulation Other, please specify _____	In-person training: 5 clinicians; Self-paced online learning: 3 clinicians; Interactive simulation: 6 clinicians
By performing virtual visits, I am able to offload in-person visits.	Strongly disagree Disagree Neutral/I don’t know Agree Strongly agree	4.5 (4.00-5.00)

### Thematic Analysis

A model emerged from the analysis that contained 3 overarching themes: (1) development of best practices, (2) barriers to scalability, and (3) establishing patient rapport. The generation of these themes suggested their applicability across participants from different disciplines, although individual clinician experiences were influenced by their subspecialty and longevity of virtual practice.

#### Theme 1: Development of Best Practices

##### Overview

Overarching the discussions was a need to develop best practices in pediatric telemedicine, including but not limited to the need to determine the ideal virtual patient, address privacy concerns, and ensure adequate physical examinations. Clinicians agreed that different disciplines could learn from one another and that, although some issues cut across disciplines, others were unique to individual subspecialties.

##### Ideal Patient

The ideal telemedicine patient was described by focus group participants as one who would experience the potential benefits of telemedicine (ie, living far from the hospital and whose parents’ capacity to take time off work was limited), is already comfortable with the clinician, and whose physical evaluation requires minimal hands-on examination. Multiple clinicians shared that it was important to thoughtfully select the patients who would gain the most value from the telemedicine experience. Clinicians who represented mental health fields additionally expressed that some patients did measurably *better* with virtual visits than with in-person visits:

We’re doing these appointments not because we expect a postoperative complication because we’ve kind of screened these patients out, but we’re doing them as a touch point to the patient so that they feel cared for and so that their perception of care is better just because we’re looking at them and we’re talking to them.

We also have a lot of patients that are coming from like South Shore or just a long ways away from the hospital, I think like all of us and it’s so nice to be able to...especially if it’s a visit where we’re kind of just checking in on like their experience on a medication or side-effects where it may only need to be like a 20-minute conversation that can happen without a two-hour drive. That feels good for everybody so I don’t think there’s been any downside to it that we’ve seen yet.

There’s a number of kids, if they are on the autism spectrum or have connection difficulties socially, it seems that like I’ve had communication is easier for them, like it’s more approachable for them. And so sometimes we’re just able to get more out of them than we would if we were in person where there’s something that is physically just...in the room it’s hard for them about connecting in person.

##### Privacy Concerns

Across disciplines, clinicians shared concerns regarding patient privacy and exchanged best practices for dealing with sensitive physical examinations:

I’ve started telling patients, just because this was on my mind about the privacy issues, etc., their comfort level, I started telling patients that I’m in my office, I’m in my private office, and nobody is going to open the door.

I do say that in the office for the older kids...we’re going to examine down here. You only do this if there’s a doctor and your parents in the room. I guess I really hadn’t said that when I’m on the telephone...that’s probably a good idea.

We also built in the support piece so probably we wanted to make sure that the patient felt comfortable and safe and so we have a social worker call them right after the visit, I contact them a week later just to make sure that they’re feeling okay with it.

##### Physical Examination

Regarding the virtual physical examination, most clinicians felt they could do a *good enough* examination for the purpose of the visit. Some clinicians expressed concerns regarding the patient’s or parent’s impression of the examination, often because of the technical issues with video equipment or lack of user experience. Clinicians also shared how they adapted their use of technology to meet the needs of their specific clinical practice:

It only took probably a handful of cases to make it obvious that we do see what we need to see very clearly. I think it’s exceeded all of our expectations for sure.

Sometimes it does lead to maybe a suboptimal exam where you’re like okay, well, I’m sure it’s fine, I can see it well enough but I’d love to see it better and it’s certainly not the same as seeing them in person; it’s just probably “good enough.” But I love leaving those with thinking this virtual visit was equivalent to my physical, to my in-person physical examination and often they do feel that way. But when the camera is jiggly or the connection’s not great, I don’t feel that way. I feel that it’s good enough, but that’s a little bit of a slippery slope if you think about it.

So yesterday I had the big sister actually get on the other side of the iPad and have the baby look at the big sister and then I said okay, I see the eyes go to the left, so I had like sister, big sister run to the left and the baby goes ooh, follows the kid, and I’m like mom, hold the head so I can see the eyes moving and it was an awesome way to do the exam. And so, then the big sister was running back and forth and the kid’s eyes are going back and forth, I’m like this is great.

#### Theme 2: Barriers to Scalability

##### Overview

Many clinicians mentioned the challenges they faced, which made them concerned about the quality and effectiveness of virtual visits. Some issues involved overcoming technical difficulties for the patients, families and clinicians, which occupied time during the visit. There were also concerns regarding privacy and how that might limit the environment in which a visit could take place.

##### Family Preparation and Education

Clinicians noted that although previsit educational materials were provided to families, these materials were not adequate, as clinicians spent a significant amount of time assisting patients and families with technical issues:

I estimate like probably 20% of my time, of my patient load is spent doing a lot of explaining of things.

I find that they’re not reading these things and so we’re trying to educate them but our education has not been effective thus far with the handouts, with the carousel screen.

That’s my problem. More than 50% of my visits are spent with 50% of my time teaching best practices and the more that I’ve realized that the best practices actually allow me to see that postoperative surgical wound better, to give that equivalent virtual experience as the in-person experience, the more frustrated I’ve become with the idea that the patients I don’t think are reading these best practices.

##### Privacy and Safety

Clinicians expressed concern that patients and families did not receive adequate information regarding the privacy and confidentiality of encounters. Some suggested changes that could be made individually and at a program level to improve patient and family knowledge of privacy restrictions around virtual visits. Clinicians expressed apprehension regarding performing sensitive examinations virtually. This was especially concerning for specialties such as urology, in which the physical examination primarily involves sensitive areas of the body:

And so to this point of preparation, we have no sort of documentation; we have no pre-visit preparation telling them these things, that your provider will be in a private spot, there will be nobody else present, etc., your privacy is guarded, we have no...guidance on this with the families at all. I think we have to be much more careful with all these things to make sure that they come away feeling really confident and safe in this experience.

So at this point the patients and the families don’t receive anything that says that you can feel safe and that any pictures obtained during the...there’s nothing like that?

Well couldn’t it be part of the carousel, even just a reminder that this is still a private appointment and that any information obtained is really part of your health record, that would be just to remind them because they have to read those things because they go around.

So I don’t know, it’s something that I think for those that do that sort of sensitive exam to really consider how we should best prepare families to do that

And these are interactions with these patients virtually with sensitive exams—we have not set any expectations, we have not set any boundaries.

##### Logistical Issues

Clinicians acknowledged issues with patients and families using technology that limited the effectiveness and impact of virtual interactions. Clinicians expressed frustration with the technology not functioning as well as they thought it should, resulting in a poor connection or an image that would make the visit difficult:

…it’s a struggle, to get—to teach them how to turn, you know, how to reverse the camera and they’re like what, and then they hang up

...sometimes physical exam is—It’s impossible either A, you can’t figure out how to focus the camera, kids moving. Their whatever, bandwidth is horrible, so you’re—it’s like this blurred image anyway. A lot of it they just can’t get it and eventually you’re just like okay, good enough. That’s it.

...the issue is like the camera is in the corner but the image is in the center of the screen. So, like if this is the baby, getting them to line up the corner with what you want to see, versus the middle

...but it’s just the optics that whatever the bandwidth is not always good enough that we can actually see enough...Nothing you can do about it. Either I have to bring them back in...Decide on how important it is.

If the bandwidth is bad there’s just nothing—you’re going to have to bring them in eventually. That’s probably the biggest limitation at the moment for me.

Well, I understand that there’s only so many limitations, I can’t call the help desk and say, hey guess what, their WI-FI is horrible

#### Theme 3: Establishing Patient Rapport

##### Overview

Clinicians acknowledged a learning curve in their ability to use telemedicine technology to provide optimal patient experience. Part of this is based on their own comfort level and confidence but is complicated by learning how to establish new ways to interact with and build rapport with families via this modality. As such, not all clinicians come away with a positive impression of the experience.

##### Clinician Confidence and Flexibility

Clinicians discussed how their own comfort, confidence, and flexibility were critical to the effective use of virtual visits:

I think it’s getting more comfortable behind the camera; it’s just I think being less stiff and sort of bringing what I bring to the bedside to the camera and in trying to remember that and not being uncomfortable with the media part of this.

They have to understand that I believe in it and that it’s working. Like in the beginning, because I wasn’t sure myself and I had to figure it out, and how am I going to talk to them about it and all this, and I no longer say this is something we’re trialing out and all that. You know, we haven’t done it before. I just say this is what we do. You’ll find it very helpful.

##### Patient Interactions

Clinicians acknowledged the importance of establishing rapport with families via virtual visits. They noted that interactions can be less natural and expressed particular anxieties about certain circumstances that virtual visits may make more challenging, such as communicating about privacy issues:

And sort of getting that whole thing in there and I think connecting in a personal way, for me, is acknowledging some of the difficulties, especially for our population and this is just such a huge undertaking for parents and families and just saying kind of hang in there, that kind of thing. So I think those personal statements from me are important

...it’s been a little weird, I’ve got to be honest, with little boys that are old enough, the mom calls them over and what happens in the office is the parents will routinely say “remember, Jimmy, only mommy, daddy and the doctor,” right, and then I say “exactly,” talk them through it, make sure they’re comfortable. On the video it’s mom saying “okay, Jimmy, remember it’s only video when it’s the doctor”

##### Clinicians’ Impression of the Virtual Interaction

Clinicians had both positive and negative impressions of the virtual interaction and their ability to establish rapport with families via virtual visits. Clinicians seemed surprised by their positive experiences. Negative experiences focused on control of the encounter:

I just feel like...that I have an ability to still connect with patients the way that I like to. I still take in information the way I do as a clinician. I do look around the room, I do look at siblings, I do look at the interaction of parents, those kinds of things, so it’s not an isolated FaceTime experience I think that you would...and I expected it to be kind of a little bit sterile or super-removed I think.

I have found that some of my patients have used this as a liberty to change the way this relationship is going to work, that all of a sudden now all the kids can run around, all of a sudden like other things can go on like the plumber coming in to fix the house at the same time as our visit. Now I know some of these things are out of control, like I get a page in the middle of a visit and I have to step out of an appointment, so it happens, but my impression is that the percentage of times that this happens is higher when they’re in the home environment versus when they are in our office environment.

## Discussion

### Principal Findings

This qualitative study of clinicians at a large, academic pediatric medical center who were initial users of telemedicine (before the COVID-19 pandemic) identified 3 key themes that are valuable to the understanding of how patient-to-provider virtual visit programs may be sustainable and generalizable for pediatric patient care in the long term. These included a need to develop best practices in pediatric telemedicine, particularly regarding patient selection, privacy and physical examination; barriers to scalability, including technical and logistical issues as well as privacy concerns; and the ability of clinicians to establish rapport with patients through virtual visits. Issues and concerns related to privacy were salient across all themes, and clinicians noted opportunities for shared learning across subspecialties.

The last 2 decades have seen a growth in the use of telemedicine, particularly for medically underserved communities [9,15-18]. Advocates for its use in pediatrics have pushed to reduce barriers as a means of expanding access to pediatric care [9]. However, addressing the need for stable funding and adequate training in technology use has been highlighted as an important priority for the forward expansion of telemedicine [9], and this study makes it apparent how essential this is for the successful implementation of telemedicine in the pediatric setting. As health care clinicians learn their clinical skills, part of their training is the development of tools and habits they will use to establish positive bonds with their patients. The clinician’s ability to interact with patients and deliver high-quality care can be described as bedside manner. The concept of *webside manner* was introduced to highlight that telemedicine interactions may require new training and learned skills in a variety of domains, including technology, to ensure the same level of clinician interaction with patients [10,19]. This qualitative study of telemedicine providers brings to light the importance of formal training in *webside manner* to optimize the virtual visit experience.

### Comparison With Prior Work

This study reflected the views of initial telemedicine users before the COVID-19 pandemic. The findings were comparable with a prior framework of early adopters, which highlights the need for clinicians to be flexible and attentive to the nonmedical aspects of patient interaction [20]. Despite the prevalence of technology in health care, clinicians in this study reported technical and logistical issues that affected the virtual visits. Families were given instructions on how to use telemedicine; however, many still had trouble or did not fully understand the instructions. In addition, some clinicians raised concerns that the video quality may not be adequate for all situations.

Similar concerns about technology and patient selection have been raised elsewhere in the literature [15,21-23]. A qualitative study among rural health clinicians in the United States also specifically identified concerns about how technology may affect personal relationships, in this case among generalists and subspecialists [16]. A qualitative study in Australia of rural and urban health care clinicians with variable levels of exposure to telemedicine identified that those with greater telemedicine experience recognized the need to be pragmatic about the risks and challenges of telemedicine as well as for ongoing technology support [24]. In this study, clinicians broadly reflected on their rapid experiential learning and had varied attitudes regarding their comfort with patient interaction. Other studies evaluating clinician attitudes around telemedicine integration into pediatric care similarly suggest that contextual factors, such as perceived usefulness of telemedicine and ease of use, may affect uptake and concern regarding the impact of technology on the patient relationship [16,25].

The 2 other prominent issues in focus group discussions were safety and privacy. Traditional health care visits take place in controlled environments that are designed to ensure safety and privacy, allowing the clinician to focus on the patient and their family. In a virtual visit, the clinician has limited control over where the patient and family is during the visit, and the patient cannot see the clinician’s surroundings. Consequently, privacy is a concern raised by both clinicians and patients, and adequate education and tools are of paramount importance [15,23,26,27]. Clinicians in this study agreed on the importance of ensuring safety and privacy, although the methods and tools used to address them varied. During the focus groups, clinicians had a real-time exchange of ideas regarding their telemedicine improvement strategies, and their engagement in learning from one another around this issue supports the need for further development of best practices in this area. Some actionable recommendations that follow from these discussions are outlined in Table 3.

**Table 3:** Actionable recommendations.

Category	Actionable recommendation	Responsible for implementation
Technology	Incorporate tools to support previsit technical testing (eg, video, audio, and connection) for both patients and clinicians Develop and use HIPAA^a^-compliant methods for patient-to-provider sharing of content (eg, photos and laboratory data) before or during the virtual visit	Institution
Environment	Ensure that the physical environment supports a private and professional virtual encounter	Clinician
Training	Create standardized patient and clinician user guides for the virtual visit platform; include major technical issues, best practices, and explanation of privacy issues Include specific physical exam guidance, depending on subspecialty, including language around privacy Share written content and links to published resources for virtual visits Include simulation for onboarding and virtual visit training with a mock, recorded virtual visit	Institution
Webside manner	Pay attention to the nonmedical aspects of the interaction (eg, eye contact) to ensure the most favorable patient experience	Clinician

^a^HIPAA: Health Insurance Portability and Accountability Act.

Although providers in this study expressed concern about the scalability of telemedicine before the COVID-19 pandemic, the pandemic reframed thinking about the potential barriers to telemedicine, such as financial concerns about reimbursement, credentialing and licensing, and medical liability [15]. These issues have been addressed by government-mandated policies in the short term and may have some residual impact on framing access to telemedicine services going forward [15,28-32]. Some pediatric settings have demonstrated the ability to rapidly scale telemedicine during COVID-19, developing novel mechanisms to connect with families that ensure privacy [33]. These success stories offer opportunities for *proof of concept* demonstrations, whereby eliminating some of the barriers has paved the way for infrastructural scaling. This may suggest that some of the logistical concerns regarding scaling expressed by clinicians in the study may be addressed by continued advances in telemedicine technology, adequate information technology support, and ongoing relaxation in these other areas.

However, it does not fully address the concerns regarding logistical issues for patients and families and aspects of privacy that are based not solely on technology but also on how clinicians are educated to interact with families via telemedicine platforms. Consistent with other studies [34], we demonstrate that ensuring long-term success with telemedicine will require an appropriate selection of patients and education for clinicians as well as patients and families. Furthermore, despite the recent need and swift implementation of telemedicine, this study’s findings, reflecting a range of specialties and professional backgrounds, suggest that enduring concerns regarding selecting the medical conditions or circumstances for which telemedicine is appropriate, privacy concerns, and the impact on the patient–clinician interaction will warrant ongoing attention to ensure that access is adequately balanced with quality [15,19,34-36]. Given the rapid expansion of telemedicine because of the COVID-19 pandemic, recognizing how to address some of these concerns is critical to ensuring that a broader range of clinician and patient experiences are optimized.

### Strengths and Limitations

This study has a number of strengths. All participants were pediatric clinicians, which provided internal consistency. Specific issues of relevance to the pediatric visit included the participation of multiple family members, control of the technology by someone other than the patient, and the challenge of managing pediatric comprehension of technology. In addition, the study was completed before the COVID-19 pandemic, which has increased telemedicine use exponentially. As such, the clinicians in our study had more control over their early telemedicine practice and the ability to choose which patients were the most appropriate for virtual visits. Therefore, the perspectives of these clinicians provide insight into the implementation lessons of telemedicine before the COVID-19 pandemic, when scaling and coverage became the priority.

Several limitations of this study should be noted. The study took place at a single pediatric institution in Massachusetts, which at the time had some of the more restrictive laws regarding telemedicine. The reimbursement structure limited the types of patients and visits that could be performed. Accordingly, most clinicians were from surgical specialties, and many of the visits being conducted were postoperative visits, as these fall under the global charge capture. However, the intent was not that study findings would reflect the full range of practice available by virtual visits but rather inform the perspectives of participants and future directions. It should be noted that one of the authors participated in the first focus group. As this author was not involved in data analysis and there were similar themes in both focus groups, we are confident that this did not introduce bias into the discourse. Finally, the focus of this study was on clinician perspective. Although not specifically a limitation of the methods, exploring the patient and family perspective of telemedicine should be an important component of future studies and can be informed by the themes identified here. In addition, further investigation with other provider groups with different types of telemedicine experience would strengthen the potential for generalizability of the study findings.

### Conclusions

It is likely that a substantial portion of clinical practice will continue to be performed virtually even after the COVID-19 pandemic. The findings of this study suggest that telemedicine curricula, including instruction on *webside manner* skills, should be incorporated into medical training. Integration of this training for all programs will be crucial to the efficacy and sustainability of this essential mode of health care delivery. Some medical school programs have already taken on this challenge [37]. As one focus group participant noted, “I think that the broader concept of webside manner should apply to all of us, kind of like what we do in medical school, PA school, nursing school, so that would be my hope in terms of future direction.” Future work may address how such training affects the patient–clinician experience and the ability to further scale telemedicine.

## Appendix

Multimedia Appendix 1 Focus group guide discussion questions.
