# Supplementary material for: Pediatric Clinicians’ Use of Telemedicine: Qualitative Interview Study
Source: JMIR Hum Factors. 2021 Dec 2;8(4):e29941. doi: 10.2196/29941 (PMC8686477; doi:10.2196/29941)
Supplement: Multimedia Appendix 1 [file humanfactors_v8i4e29941_app1.docx]

| ***Category*** | ***Questions*** |
| --- | --- |
| **Experience** | - *What were your expectations going into virtual visits? How did these change after you started offering them?* - *What are some good/bad experiences you’ve had, and what made them good/bad?* |
| **Preparedness** | - *What things did you notice after go-live that you did not receive optimal education on?* - *What would your advice be to a new clinician beginning to offer virtual visits?* |
| **Communication and establishing rapport** | - *How do you establish rapport with the patient/family who is located remotely?* - *How you do you think that the virtual visit platform affects your ability to communicate and counsel families about their child’s medical situation?* - *Do you use any nonverbal cues to enhance communication with the patient/family?* |
| **Effectiveness of visit** | - *From a clinical assessment standpoint: what do you feel like you’re not getting/able to do during a virtual visit and how do you address that?* - *What parts of the history or physical exam have you changed as you completed more virtual visits?* - *Are there things that if the patient were educated or instructed, could they help participate in doing a clinical assessment virtually?* - *Have you done anything additional/innovative to try to address those limitations?* - *What best-practices on the patient end might make virtual visits more effective?* - *Are there any visits that posed particular challenges for you? Or for the patient? How did you solve them?* |

**Supplemental Table 1. Focus Group Guide Discussion Questions.**
